# Global and regional burden of attributable and associated bacterial antimicrobial resistance avertable by vaccination: modelling study

**DOI:** 10.1136/bmjgh-2022-011341

**Published:** 2023-07-05

**Authors:** Chaelin Kim, Marianne Holm, Isabel Frost, Mateusz Hasso-Agopsowicz, Kaja Abbas

**Affiliations:** 1 Policy & Economic Research (PER) Department, International Vaccine Institute, Seoul, Korea (the Republic of); 2 Infectious Diseases, The Novo Nordisk Foundation, Copenhagen, Denmark; 3 Department of Immunization, Vaccines and Biologicals (IVB), World Health Organization, Geneva, Switzerland; 4 Department of Infectious Disease Epidemiology, London School of Hygiene & Tropical Medicine, London, UK

**Keywords:** Immunisation, Vaccines

## Abstract

**Introduction:**

Antimicrobial resistance (AMR) is a global health threat with 1.27 million and 4.95 million deaths attributable to and associated with bacterial AMR, respectively, in 2019. Our aim is to estimate the vaccine avertable bacterial AMR burden based on existing and future vaccines at the regional and global levels by pathogen and infectious syndromes.

**Methods:**

We developed a static proportional impact model to estimate the vaccination impact on 15 bacterial pathogens in terms of reduction in age-specific AMR burden estimates for 2019 from the Global Research on Antimicrobial Resistance project in direct proportion to efficacy, coverage, target population for protection, and duration of protection of existing and future vaccines.

**Results:**

The AMR burden avertable by vaccination in 2019 was highest for the WHO Africa and South-East Asia regions, for lower respiratory infections, tuberculosis, and bloodstream infections by infectious syndromes, and for *Mycobacterium tuberculosis* and *Streptococcus pneumoniae* by pathogen. In the baseline scenario for vaccination of primary age groups against 15 pathogens, we estimated vaccine-avertable AMR burden of 0.51 (95% UI 0.49–0.54) million deaths and 28 (27–29) million disability-adjusted life-years (DALYs) associated with bacterial AMR, and 0.15 (0.14–0.17) million deaths and 7.6 (7.1–8.0) million DALYs attributable to AMR globally in 2019. In the high-potential scenario for vaccination of additional age groups against seven pathogens, we estimated vaccine-avertable AMR burden of an additional 1.2 (1.18–1.23) million deaths and 37 (36–39) million DALYs associated with AMR, and 0.33 (0.32–0.34) million deaths and 10 (9.8–11) million DALYs attributable to AMR globally in 2019.

**Conclusion:**

Increased coverage of existing vaccines and development of new vaccines are effective means to reduce AMR, and this evidence should inform the full value of vaccine assessments.

**WHAT IS ALREADY KNOWN ON THIS TOPIC**

- There is some evidence on the impact of vaccines against *Haemophilus influenzae* type b, rotavirus, *Streptococcus pneumoniae*, *Salmonella typhi* and influenza on antimicrobial resistance (AMR) in specific settings.

**WHAT THIS STUDY ADDS**

- To our knowledge, this is the first study to estimate attributable and associated bacterial AMR burden avertable by vaccination against 15 bacterial pathogens for a combined set of existing and new vaccines in the pipeline by pathogen, infectious syndrome and region.
- The AMR burden avertable by vaccination in 2019 was highest for the WHO Africa and South-East Asia regions, for lower respiratory infections, tuberculosis and bloodstream infections by infectious syndromes, and for *Mycobacterium tuberculosis* and *Streptococcus pneumoniae* by pathogen.

**HOW THIS STUDY MIGHT AFFECT RESEARCH, PRACTICE OR POLICY**

- Our model-based projections facilitate evidence-based decision-making for scaling up of existing vaccines to regions in most need with higher AMR burden and prioritise development of new vaccines with high potential for lowering AMR burden by pathogen, infectious syndrome and region.
- Our study contributes to the WHO-led value attribution framework for vaccines against AMR, and specifically to the criterion focused on vaccine averted AMR health burden.

## Introduction

Since the discovery of penicillin in 1928, antimicrobials have been used to treat bacteria, fungi, parasites and viruses, saving countless lives.^1^ However, antimicrobial resistance (AMR) is a growing global public health threat in the 21st century.^2^ Resistance occurs through pathogen evolution, either naturally over time or acquired by the use of antimicrobial drugs, which render these drugs ineffective and increase the risk of morbidity and mortality. While access to antimicrobial drugs in low-income and middle-income countries to treat infections continues to be a challenge, misuse and overuse of antimicrobials along with lack of access to clean water, sanitation and hygiene and effective infection prevention and control measures have fuelled the emergence and spread of AMR globally. The UK government commissioned review on AMR in 2014 projected that if AMR is not controlled, it would lead to significant impact on health with 10 million AMR-related deaths annually and macroeconomic consequences with a cumulative economic loss of US$100 trillion by 2050.^3^

Vaccination, when used in conjunction with other preventive measures, has the potential to significantly reduce AMR transmission through several pathways.^4 5^ First, vaccination has a direct influence on the health burden of AMR by preventing the emergence and transmission of drug-resistant and drug-sensitive infections, and the associated antibiotic use. Second, vaccines have an indirect influence by reducing resistant infections in unvaccinated populations through herd immunity. Third, vaccination can prevent infections where antimicrobials are not indicated but often wrongly prescribed, such as primary viral infections, thereby reducing misuse and overuse of antimicrobials. Fourth, vaccines can also reduce the use of antimicrobials to treat secondary bacterial infections caused by viral diseases. Finally, vaccines can give longer-term health benefits in preventing infections and resistance to vaccines is rarely observed.^6^

The Global Research on Antimicrobial Resistance (GRAM) project estimated the deaths and disability-adjusted life-years (DALYs) attributable to and associated with resistance by replacing all drug-resistant infections with susceptible infection or no infection, respectively. It estimated that 1.27 (95% UI 0.91–1.7) million deaths and 47.9 (35–64) million DALYs were attributable to bacterial AMR and 4.95 (3.6–6.6) million deaths and 192 (146–248) million DALYs were associated with bacterial AMR in 2019.^7^ Despite the significant potential impact of vaccination in lowering AMR, evidence is limited due to the methodological difficulties and challenges in obtaining data on the health burden associated with AMR in order to calculate this impact.^8–10^ Such evidence will be valuable to inform improvements in the coverage of existing vaccines and prioritise research and development of new vaccines.

To address this evidence gap, our aim is to analyse the findings from the GRAM project and estimate the vaccine-avertable bacterial AMR burden based on the profiles of existing and future vaccines by pathogen and infectious syndromes at the regional and global levels in 2019. Such pan-pathogen analyses using standardised approaches are critical to inform vaccine development, funding, introduction and use. They also inform the WHO-led value attribution framework for vaccines against AMR,^11^ which includes five criteria: (1) vaccine averted AMR health burden, (2) vaccine averted AMR economic burden, (3) vaccine averted antibiotic use, (4) sense of urgency to develop antimicrobial approaches and (5) pathogen impact on equity and social justice. Our study contributes to the first criterion—vaccine-averted AMR health burden.

## Methods

### AMR burden data

We used the bacterial AMR burden estimates from the GRAM project which provided data for age-specific deaths and DALYs associated with and attributable to AMR by pathogen, infectious syndrome and region for 2019.^7 12^ These comprehensive estimates of bacterial AMR burden were based on statistical predictive modelling of data from systematic reviews, surveillance systems, hospital systems and other sources to generate estimates for 23 pathogens and 88 pathogen-drug combinations for 204 countries in 2019. The AMR burden estimates for *Neisseria gonorrhoeae* include only morbidity and no mortality.

Two sets of estimates are presented—burden attributable to AMR, that is, deaths and DALYs that could be averted if all drug-resistant infections would be replaced by drug-sensitive infections; and burden associated with AMR, that is deaths and DALYs that could be averted if all drug-resistant infections would be replaced by no infections. As vaccines prevent drug-resistant and drug-susceptible burden, we infer that the associated AMR burden is the appropriate metric for measuring the impact of vaccination on AMR burden.

### Vaccine profiles

We focused our analysis on 15 pathogens*—Acinetobacter baumannii*, *Enterococcus faecium*, *Escherichia coli*, Group A *Streptococcus*, *Haemophilus influenzae*, *Klebsiella pneumoniae*, *Mycobacterium tuberculosi*s, *Neisseria gonorrhoeae*, non-typhoidal *Salmonella*, *Pseudomonas aeruginosa*, *Salmonella paratyphi*, *Salmonella typhi*, *Shigella* spp, *Staphylococcus aureus* and *Streptococcus pneumoniae*. We selected pathogens that are part of the WHO evaluation of the value of vaccines in preventing AMR. We used vaccine profiles (see table 1), which comprise the vaccine target population, efficacy, coverage, duration of protection and disease presentation prevented. For the existing vaccines against *H. influenzae* type b, *S. pneumoniae* and *S*. typhi, the vaccine profiles expand coverage of the current vaccines in order to meet the strategic priority on coverage and equity of Immunisation Agenda 2030.^13^ For vaccines that are not yet available, hypothetical profiles were developed based on preferred product characteristics (PPCs),^14^ target product profiles (TPPs), attributes of advanced vaccine candidates and expert consultations with WHO working groups, PATH and pathogen experts. Some pathogens have multiple disease presentations and would require different vaccines to prevent different disease presentations. As such, these pathogens have more than one vaccine profile.

**Table 1:** Vaccine profiles

Pathogen	Disease presentation	Vaccine			Vaccination scenarios (age of vaccination)		Justification
		Efficacy (%)	Coverage (%)	Duration of protection	Baseline scenario	High-potential scenario	
*Acinetobacter baumannii*—BSI*	BSI	70	70	5 years	6 weeks, elderly age group with highest burden	All age groups	Hypothetical vaccine based on expert opinion
*A. baumannii*—all	All (Bacterial skin infections, BSI, cardiac infections, LRI and thorax infections, UTI)	70	70	5 years	6 weeks, elderly age group with highest burden	All age groups	Hypothetical vaccine based on expert opinion
*Enterococcus faecium*	All (bone and joint infections, BSI, cardiac infections, intra-abdominal infections, UTI)	70	70	5 years	6 weeks, elderly age group with highest burden	All age groups	Hypothetical vaccine based on expert opinion
*Enterotoxigenic Escherichia coli*	Diarrhoea	60	70	5 years	6 months	–	WHO Preferred Product Characteristics and Expert opinion and Advanced candidate
Extraintestinal Pathogenic *E. coli* (ExPEC)—BSI*	BSI	70	70	5 years	6 weeks, elderly age group with highest burden	All age groups	Hypothetical vaccine based on expert opinion
ExPEC—UTI*	UTI	70	70	5 years	6 weeks, elderly age group with highest burden	All age groups	Hypothetical vaccine based on expert opinion
*E. coli*—non diarrheagenic	Bacterial skin infections, bone and joint infections, BSI, cardiac infections, CNS infections, intra-abdominal infections, LRI and thorax infections and UTI	70	70	5 years	6 weeks, elderly age group with highest burden	All age groups	Hypothetical vaccine based on expert opinion
Group A *Streptococcus*	All (bacterial skin infections, bone and joint infections, BSI, cardiac infections)	70	70	5 years	6 weeks	–	WHO Preferred Product Characteristics and Expert opinion
*Haemophilus influenzae* type B	All (CNS infections, LRI and thorax infections)	59; 92; 93† (69 for LRI)	90	5 years	6, 10, 14 weeks	–	Existing vaccine
*Klebsiella pneumoniae*—BSI	BSI	70	70	6 months	0 weeks (maternal)	–	Hypothetical vaccine based on expert opinion
*K. pneumoniae*—all	All (bacterial skin infections, bone and joint infections, BSI, cardiac infections, CNS infections, intra-abdominal infections, LRI and thorax infections, UTI)	70	70	5 years	6 weeks, elderly age group with highest burden	All age groups	Hypothetical vaccine based on expert opinion
*Mycobacterium tuberculosis*—M72*	Tuberculosis	50	70	10 years	10 years+boost every 10 years	–	WHO Preferred Product Characteristics and Expert opinion and Advanced candidate
*M. tuberculosis*—Improved	Tuberculosis	80	70	10 years	0 weeks (at birth) + boost every 10 years	–	WHO Preferred Product Characteristics and Expert opinion
*Neisseria gonorrhoeae*	Gonorrhoea	70	70	10 years	15 years	–	WHO Preferred Product Characteristics and Expert opinion
Non-typhoidal *Salmonella*	All (BSI, cardiac infections, diarrhoea, typhoid, paratyphoid and iNTS)	80	70	5 years	6 weeks, 9 months	–	Hypothetical vaccine based on expert opinion
*Pseudomonas aeruginosa*	BSI, LRI and thorax infections	70	70	5 years	6 weeks, elderly age group with highest burden	All age groups	Hypothetical vaccine based on expert opinion
*Salmonella paratyphi*	Typhoid, paratyphoid and iNTS	70	70	5 years	9 months	–	Hypothetical vaccine based on expert opinion
*Salmonella typhi*	All (BSI, cardiac infections, typhoid, paratyphoid and iNTS)	85	70	15 years	9 months	–	Existing vaccine and Expert opinion
*Shigella*	All (diarrhoea)	60	70	5 years	6 months	–	WHO Preferred Product Characteristics and Expert opinion and Advanced candidate
*S. aureus*	All (Bacterial skin infections, bone and joint infections, BSI, cardiac infections, CNS infections, intra-abdominal infections, LRI and thorax infections, UTI)	60	70	5 years	6 weeks, elderly age group with highest burden	All age groups	Hypothetical vaccine based on expert opinion
*Streptococcus pneumoniae*	BSI, CNS infections, cardiac infections, LRI	29; 58; 58† (27 for LRI)	90	5 years	6, 10, 14 weeks	6, 10, 14 weeks, elderly age group with highest burden	Existing vaccine
*S. pneumoniae*—Improved*	BSI, CNS infections, Cardiac infections, LRI	70 (50 for LRI)	90	5 years	6 weeks	6 weeks, elderly age group with highest burden	Hypothetical vaccine based on expert opinion

Product characteristics for efficacy and duration of protection for vaccine-derived immunity, and coverage and target population (age of vaccination) for current and future vaccines against bacterial pathogens. The baseline scenario includes 15 pathogens, and the high-potential scenario includes a subset of 7 pathogens.

*The effects of these vaccines were not added to the aggregated impact of vaccination on AMR burden by region and by infectious syndrome.

†Efficacy corresponding to first, second and third doses respectively.

AMR, antimicrobial resistance; bacterial skin infections, bacterial infections of the skin and subcutaneous systems; Bone and joint infections, infections of bones, joints, and related organs; BSI, bloodstream infections; cardiac infections, endocarditis and other cardiac infections; CNS, central nervous system; CNS infections, meningitis and other bacterial central nervous system infections; intra-abdominal infections, peritoneal and intra-abdominal infections; iNTS, invasive non-typhoidal Salmonella spp; LRI, lower respiratory infection; LRI and thorax infections, lower respiratory infections and all related infections in the thorax; typhoid, paratyphoid, and iNTS, typhoid fever, paratyphoid fever, and invasive non-typhoidal Salmonella spp; UTI, urinary tract infections and pyelonephritis; UTI, urinary tract infection.

### Modelling process

**Figure 1:**
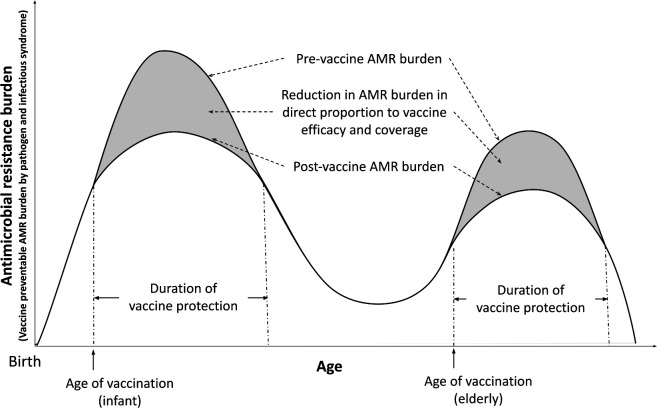
Vaccine impact on antimicrobial resistance model. Static proportional impact model to estimate the reduction in pre-vaccine AMR burden after vaccination in direct proportion to efficacy, coverage, target population for protection, and duration of protection of existing and potential future vaccines. The AMR burden among the infants could be higher or lower than the AMR burden among the elderly depending on the pathogen. For example, AMR burden for *Streptococcus pneumoniae* and *Haemophilus influenzae* are higher among the infants compared to the elderly, while AMR burden for *Staphylococcus aureus* and *Acinetobacter baumannii* are lower among the infants compared to the elderly.

We developed a static proportional impact model (see figure 1) to estimate the vaccination impact in terms of reduction in age-specific AMR burden estimates for 2019 from the GRAM project. We estimated a counterfactual prevaccination scenario for diseases with current vaccinations and adjusted for disease type specification before applying the vaccine impact. We calculated the reduction in prevaccine AMR burden after vaccination in direct proportion to efficacy, coverage, target population for protection, and duration of protection of existing and potential future vaccines.^15^

For ages that lie within the duration of protection since the time of vaccination:

AMR burden averted at age i=AMR burden at age i prevaccination × vaccine efficacy × vaccine coverage.

### Scenarios

We estimated vaccine avertable deaths and DALYs attributable to and associated with AMR by region, infectious syndrome and pathogen with 95% uncertainty intervals (UIs) for two scenarios—baseline scenario (for 15 pathogens) for primary vaccination of specific age groups, and high-potential scenario (for a subset of 7 pathogens) that includes additional age groups at risk of infection based on expert opinion.

Vaccine profiles with the corresponding product characteristics for efficacy and duration of protection for vaccine-derived immunity, and coverage and target population for the baseline and high-potential scenarios are described in table 1. In the baseline scenario, we estimated the vaccine avertable burden from the age of vaccination under the assumption that vaccine-derived immunity would sustain for the duration of protection of the corresponding vaccines. We did not consider vaccine waning dynamics due to limited evidence. For pathogens with a highly uncertain vaccine target population or feasibility of vaccine delivery, we estimated an additional high-potential scenario which assumed that individuals at risk (including additional age groups at risk) would be vaccinated to protect against corresponding disease presentations. This was applicable to vaccines against *A. baumannii*, *E. faecium*, Extraintestinal Pathogenic *E. coli* (ExPEC), *K. pneumoniae (all syndromes*), *P. aeruginosa* and *S. aureus*. For *S. pneumoniae*, we explored the high-potential scenario by administering a vaccine to an elderly population with the highest disease burden.

### Uncertainty analysis

We conducted a Monte Carlo simulation of 400 runs (sufficient for results to converge) to propagate the uncertainty in the AMR burden, vaccine efficacy and coverage through the model simulations to estimate the uncertainty in our projected outcomes of vaccination impact. We provide summary estimates in terms of vaccine-avertable deaths and DALYs attributable to and associated with AMR by region, infectious syndrome and pathogen with 95% UIs for the baseline and high-potential scenarios. Additional details on the modelling process, scenarios and uncertainty analysis are provided in online supplemental appendix A1.

10.1136/bmjgh-2022-011341.supp1 Supplementary data

### Patient and public involvement

We analysed anonymised secondary data in our study. The data analysed originated from the GRAM project, and they were analysed in aggregate. As a result, it was not appropriate or possible to involve patients or the public in the design, or conduct, or reporting, or dissemination plans of our research. The public will benefit from the findings of our study as our model-based projections facilitate evidence-based decision-making for scaling up of existing vaccines to regions in most need with higher AMR burden and prioritise development of new vaccines with high potential for lowering AMR burden by pathogen, infectious syndrome and region.

### Data availability and code repository

We conducted our analysis using the R (version 4.2.3) programming language for statistical computing,^16^ and the repository for the data and software code of this modelling study are publicly accessible at https://github.com/vaccine-impact/vaccine_amr and Dryad open data publishing platform.^17^

## Results

### Vaccine impact on global AMR burden

At the global level in 2019 for the baseline scenario, we estimated that vaccines against the 15 pathogens (analysed in this study) could avert 0.51 (95% UI 0.49–0.54) million deaths and 28 (27–29) million DALYs associated with AMR, and 0.15 (0.14–0.17) million deaths and 7.6 (7.1–8.0) million DALYs attributable to AMR. In the high-potential scenario, we estimated that vaccines against a subset of 7 pathogens could avert an additional 1.2 (1.18–1.23) million deaths and 37 (36–39) million DALYs associated with AMR, and 0.33 (0.32–0.34) million deaths and 10 (9.8–11) million DALYs attributable to AMR globally in 2019.

### Vaccine impact on AMR burden by pathogen

**Figure 2:**
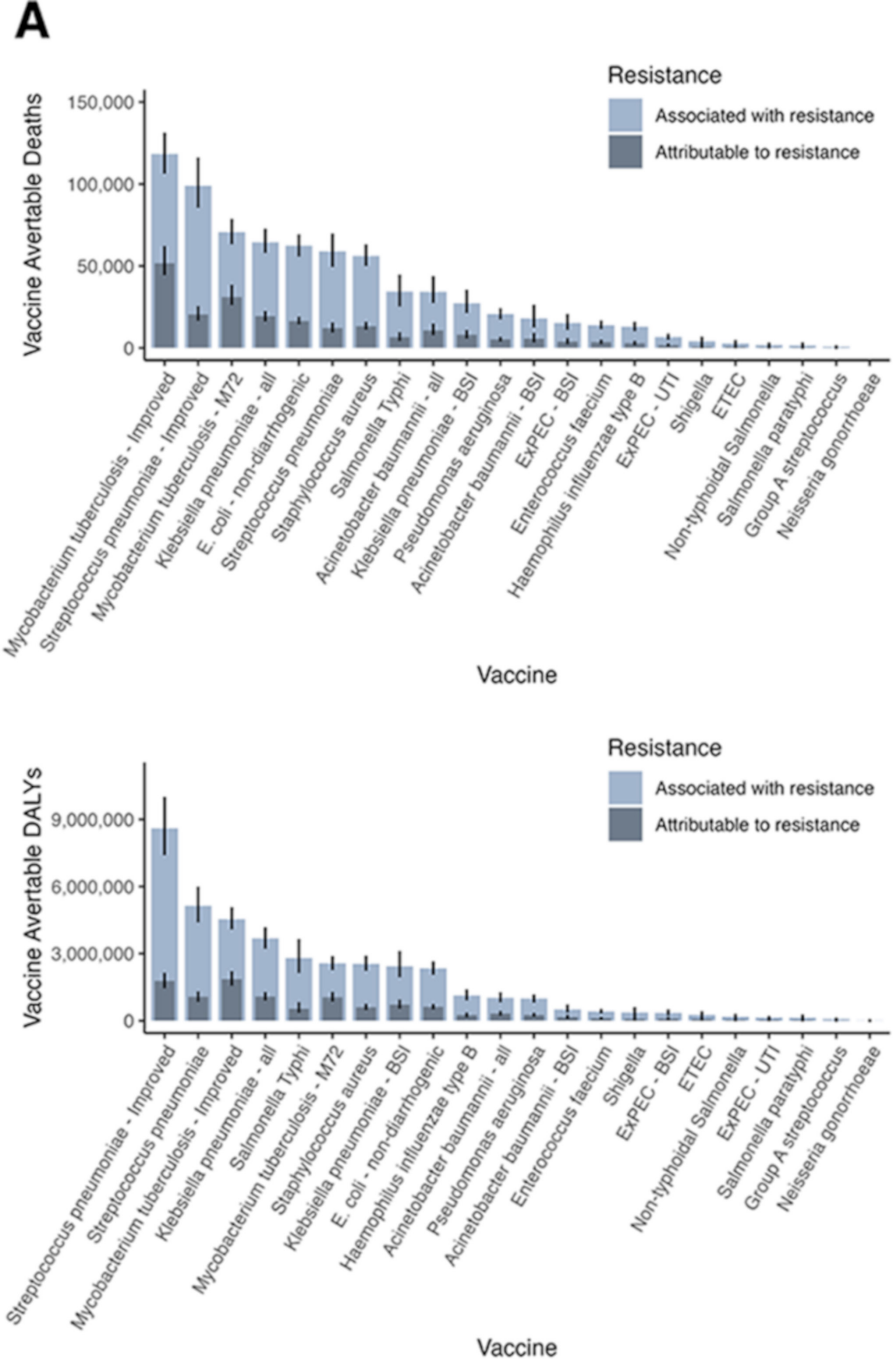

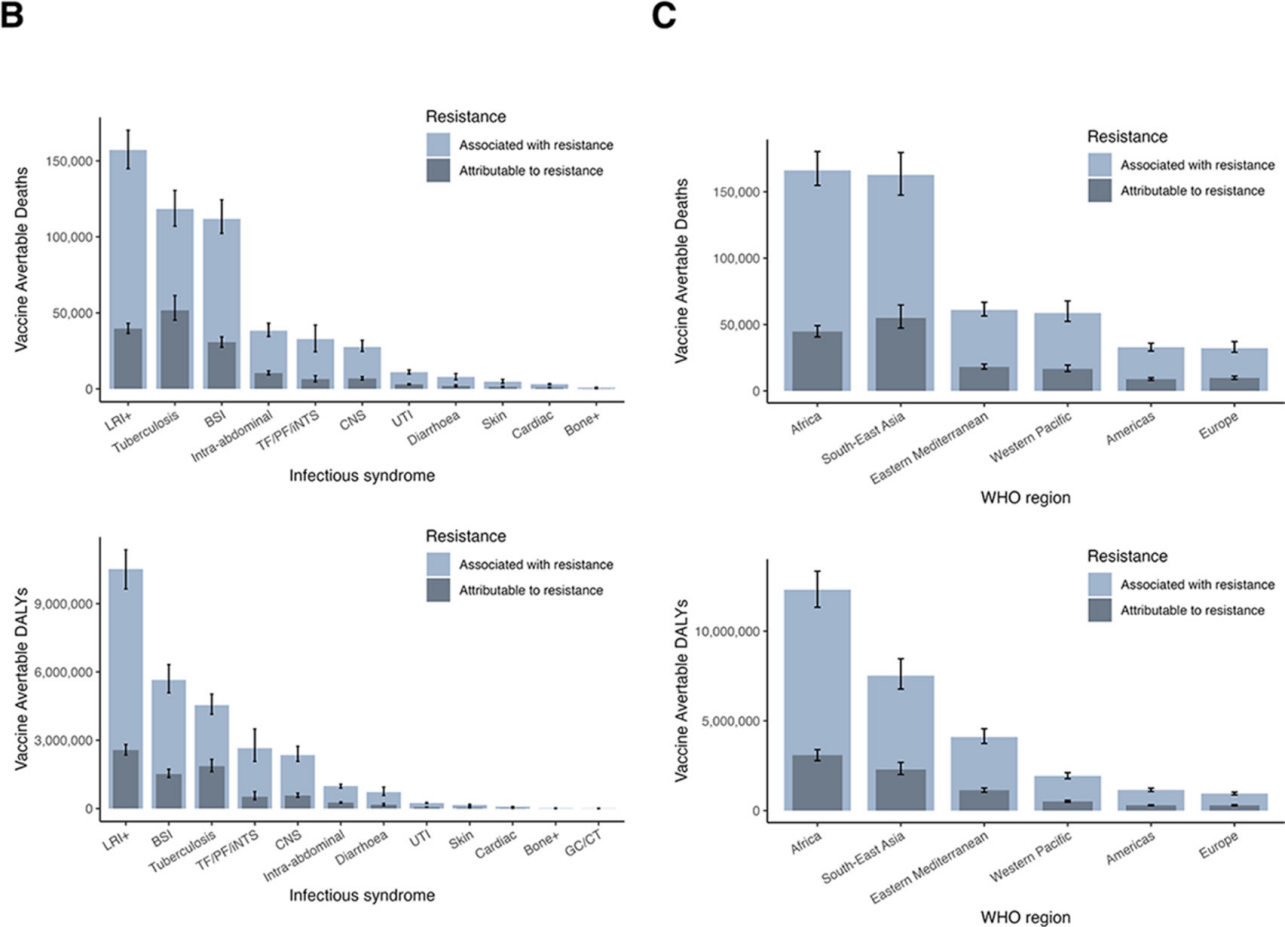
Vaccine impact on AMR burden by (pathogen-specific) vaccine profile, infectious syndrome, and region. The estimates (median and 95% uncertainty intervals) of the vaccine avertable deaths attributable to and associated with bacterial antimicrobial resistance in 2019 were aggregated by (pathogen-specific) vaccine profile, infectious syndrome, and WHO region in the baseline scenario. (Bone+ = infections of bones, joints, and related organs; BSI = bloodstream infections; cardiac = endocarditis and other cardiac infections; CNS = meningitis and other bacterial CNS infections; intra-abdominal = peritoneal and intra-abdominal infections; LRI+ = lower respiratory infections and all related infections in the thorax; skin = bacterial infections of the skin and subcutaneous systems; TF–PF–iNTS = typhoid fever, paratyphoid fever, and invasive non-typhoidal Salmonella spp; UTI = urinary tract infections and pyelonephritis).

Figure 2A and table 2A present the vaccine avertable burden attributable to and associated with AMR in 2019 for each of the pathogen-specific vaccine profiles at the global level for the baseline scenario. For pathogens with licensed vaccines, we estimated that vaccination against *S. pneumoniae* at 2019 coverage levels averted 44 (37–52) thousand deaths and 3.8 (3.3–4.5) million DALYs associated with AMR in 2019. By reaching the WHO recommended coverage level of 90% globally, 59 (50–69) thousand deaths and 5.1 (4.5–5.9) million DALYs associated with AMR could have been averted in 2019. Expanding the coverage to elderly populations would increase the vaccination impact to avert 71 (63–81) thousand deaths. We estimated that vaccination against *H. influenzae* at 2019 coverage levels averted 11 (9.7–13) thousand deaths and 0.98 (0.85–1.2) million DALYs associated with AMR in 2019. At 90% coverage globally, 13 (11–15) thousand deaths and 1.1 (0.96–1.3) million DALYs associated with AMR could have been averted. We estimated that wider introduction and scale-up of vaccination against *S*. *typhi* could have averted 34 (26–44) thousand deaths and 2.8 (2.2–3.6) million DALYs associated with AMR in 2019.

**Table 2:** Vaccine impact on AMR burden by vaccine profile

Pathogen	Disease presentation	Vaccine avertable deaths (median and 95% UI)		Vaccine avertable DALYs (median and 95% UI)	
		Associated with resistance	Attributable to resistance	Associated with resistance	Attributable to resistance
**(A) Baseline scenario**					
*Acinetobacter baumannii*—BSI	BSI	18 060 (13 305–25 668)	5 723 (4 142–8 442)	504 850 (411 310–667 559)	159 560 (123 642–211 247)
*A. baumannii*—all	All (bacterial skin infections, BSI, cardiac infections, LRI and thorax infections, UTI)	34 327 (28 241–43 094)	10 799 (8 651–14 129)	1 023 981 (875 469–1 219 073)	317 946 (268 359–382 502)
*Enterococcus faecium*	All (bone and joint infections, BSI, cardiac infections, intra-abdominal infections, UTI)	13 933 (12 268–16 025)	3 641 (3 094–4 469)	414 380 (363 986–472 183)	105 353 (91 731–121 159)
Enterotoxigenic *Escherichia coli*	Diarrhoea	2 779 (2 043–4 136)	784 (545–1 094)	257 376 (181 122–366 874)	69 078 (49 462–97 560)
Extraintestinal Pathogenic *Escherichia coli* (ExPEC)—BSI	BSI	15 316 (11 794–19 992)	3 938 (3 060–5 348)	348 547 (284 583–452 346)	89 166 (72 750–112 609)
ExPEC—UTI	UTI	6 727 (5 659–7 934)	1 787 (1 469–2 172)	140 036 (123 597–158 719)	37 240 (31 367–43 345)
*E. coli*—non-diarrheagenic	All (bacterial skin infections, bone and joint infections, BSI, cardiac infections, CNS infections, intra-abdominal infections, LRI and thorax infections and UTI)	62 424 (56 454–68 555)	16 405 (15 090–18 344)	2 342 931 (2 112 939–2 596 506)	619 287 (558 392–687 012)
Group A *Streptococcus*	All (bacterial skin infections, bone and joint infections, BSI, cardiac infections)	792 (643–998)	82 (55–130)	69 213 (55 881–87 845)	6 183 (3 872–11 337)
*Haemophilus influenzae* type B	All (CNS infections, LRI and thorax infections)	13 027 (11 058–15 180)	2 946 (2 412–3 622)	1 127 552 (961 297–1 347 824)	251 515 (199 906–311 346)
*Klebsiella pneumoniae*—BSI	BSI	27 333 (22 045–34 905)	8 116 (6 508–10 273)	2 434 932 (1 996 554–3 060 924)	714 363 (584 958–887 553)
*K. pneumoniae*—all	All (bacterial skin infections, bone and joint infections, BSI, cardiac infections, CNS infections, Intra-abdominal infections, LRI and thorax infections, UTI)	64 484 (58 747–72 028)	19 397 (16 971–21 761)	3 685 059 (3 274 095–4 127 836)	1 080 967 (954 038–1 225 072)
*Mycobacterium tuberculosis*—M72	Tuberculosis	70 704 (64 053–77 951)	31 040 (26 956–37 850)	2 567 640 (2 320 786–2 833 586)	1 047 910 (908 019–1 230 409)
*M. tuberculosis*—improved	Tuberculosis	118 316 (107 061–130 567)	51 675 (45 223–61 401)	4 542 790 (4 143 045–5 022 950)	1 860 715 (1 621 063–2 160 554)
*Neisseria gonorrhoeae*	gonorrhoea	not applicable	not applicable	8 917 (6 929–11 667)	459 (341–596)
Non-typhoidal Salmonella	All (BSI, cardiac infections, diarrhoea, typhoid, paratyphoid, and iNTS)	1 820 (1 412–2 624)	396 (290–618)	178 321 (133 842–253 309)	34 794 (24 808–52 983)
*Pseudomonas aeruginosa*	BSI, LRI and thorax infections	20 700 (18 148–23 443)	5 314 (4 633–6 081)	990 137 (859 997–1 120 193)	254 648 (222 548–295 151)
*Salmonella paratyphi*	Typhoid, paratyphoid and iNTS	1 463 (853–2 793)	301 (149–637)	127 891 (74 564–224 337)	25 868 (14 388–57 005)
*Salmonella typhi*	All (BSI, cardiac infections, typhoid, paratyphoid and iNTS)	34 478 (26 029–44 037)	6 630 (5 022–8 959)	2 799 895 (2 192 814–3 598 222)	534 312 (402 760–772 211)
Shigella	All (diarrhoea)	4 133 (2 765–6 132)	860 (545–1 557)	369 238 (242 138–552 960)	76 229 (48 803–136 638)
*Staphylococcus aureus*	All (bacterial skin infections, bone and joint infections, BSI, cardiac infections, CNS infections, intra-abdominal infections, LRI and thorax infections, UTI)	56 141 (50 768–62 454)	13 322 (11 924–15 169)	2 544 277 (2 287 576–2 854 546)	601 157 (529 162–697 354)
*Streptococcus pneumoniae*	BSI, CNS infections, cardiac infections, LRI	58 922 (50 170–69 048)	12 179 (10 178–14 772)	5 138 513 (4 453 672–5 943 041)	1 069 634 (892 728–1 256 886)
*S. pneumoniae*—improved	BSI, CNS infections, cardiac infections, LRI	98 987 (86 231–115 406)	20 415 (17 330–24 803)	8 606 730 (7 457 713–9 953 947)	1 781 930 (1 511 883–2 089 464)
**(B) High-potential scenario**					
*Acinetobacter baumannii*—BSI	BSI	116 141 (105 342–128 342)	36 641 (33 081–41 272)	3 463 881 (3 167 174–3 769 823)	1 081 836 (992 261–1 201 335)
*A. baumannii*—all	All (bacterial skin infections, BSI, cardiac infections, LRI and thorax infections, UTI)	216 584 (201 748–231 987)	67 905 (63 384–73 535)	6 018 518 (5 653 363–6 337 590)	1 854 005 (1 728 332–1 985 653)
*Enterococcus faecium*	All (bone and joint infections, BSI, cardiac infections, intra-abdominal infections, UTI)	100 814 (95 339–105 798)	26 342 (24 611–28 209)	2 727 684 (2 594 918–2 873 565)	699 956 (657 337–742 297)
ExPEC—BSI	BSI	103 016 (93 650–114 889)	26 551 (24 078–29 292)	2 664 329 (2 471 634–2 888 329)	698 896 (646 274–764 365)
ExPEC—UTI	UTI	49 669 (46 732–52 824)	13 003 (12 189–13 885)	1 079 376 (1 029 529–1 140 334)	287 866 (271 982–306 681)
*E. coli*—non-diarrhogenic	Bacterial skin infections, bone and joint infections, BSI, cardiac infections, CNS infections, intra-abdominal infections, LRI and thorax infections and UTI	389 043 (373 393–404 859)	102 352 (97 917–106 919)	12 648 212 (12 044 182–13 489 274)	3 375 286 (3 163 077–3 641 542)
*Klebsiella pneumoniae*—all	All (bacterial skin infections, Bone and joint infections, BSI, cardiac infections, CNS infections, intra-abdominal infections, LRI and thorax infections, UTI)	321 242 (308 878–335 698)	97 026 (92 013–102 088)	13 709 546 (12 834 241–14 723 484)	4 068 201 (3 801 569–4 425 285)
*Pseudomonas aeruginosa*	BSI, LRI and thorax infections	118 966 (113 054–125 950)	30 495 (28 728–32 634)	4 821 442 (4 495 854–5 257 746)	1 237 497 (1 149 086–1 347 794)
*Staphylococcus aureus*	All (bacterial skin infections, bone and joint infections, BSI, cardiac infections, CNS infections, intra-abdominal infections, LRI and thorax infections, UTI)	319 112 (307 397–331 431)	76 796 (73 583–80 782)	10 579 419 (10 085 244–11 164 417)	2 499 704 (2 357 371–2 664 681)
*S. pneumoniae*	BSI, CNS infections, cardiac infections, LRI	71 343 (62 610–81 314)	14 728 (12 661–17 487)	5 324 115 (4 648 050–6 118 739)	1 107 245 (930 760–1 296 970)
*S. pneumoniae*—improved	BSI, CNS infections, cardiac infections, LRI	118 645 (103 983–135 056)	24 471 (20 943–28 957)	8 980 361 (7 882 947–10 255 670)	1 848 190 (1 559 623–2 190 715)

The estimates (median and 95% UI) of the vaccine avertable deaths and DALYs attributable to and associated with bacterial AMR in 2019 were aggregated by vaccine profile for the baseline (A) and high-potential (B) scenarios.

AMR, antimicrobial resistance; bacterial skin infections, bacterial infections of the skin and subcutaneous systems; Bone and joint infections, infections of bones, joints, and related organs; BSI, bloodstream infections; cardiac infections, endocarditis and other cardiac infections; CNS, central nervous system; CNS infections, meningitis and other bacterial central nervous system infections; DALYs, disability-adjusted life-years; intra-abdominal infections, peritoneal and intra-abdominal infections; LRI, lower respiratory infection; LRI and thorax infections, lower respiratory infections and all related infections in the thorax; typhoid, paratyphoid, and iNTS, typhoid fever, paratyphoid fever, and invasive non-typhoidal Salmonella spp; UI, uncertainty interval; UTI, urinary tract infections and pyelonephritis.

For pathogens with hypothetical vaccine profiles (developed by experts or provided in PPCs), we estimated that a vaccine against *M. tuberculosis* that meets WHO’s PPC criteria of 80% efficacy, given to infants, with lifelong immunity or boosting, would have averted 0.12 (0.11–0.13) million deaths and 4.5 (4.1–5.0) million DALYs associated with AMR. An improved vaccine against *S. pneumoniae* (70% efficacy against bloodstream infections (BSI), meningitis and other bacterial central nervous system infections, 50% efficacy against lower respiratory infection (LRI) and all related infections in the thorax, given to 90% of infants at 6 weeks of life) would have a relatively highest impact by averting 99 (86–115) thousand deaths and 8.6 (7.5–10) million DALYs associated with AMR in 2019. An M72-like vaccine against *M. tuberculosis* given to adolescents and older populations with lifelong immunity or boosting would avert 71 (64–78) thousand deaths and 2.6 (2.3–2.8) million DALYs associated with AMR. A vaccine against all disease presentations of *K. pneumoniae* infection given to infants and elderly populations would avert 64 (59–72) thousand deaths and 3.7 (3.3–4.1) million DALYs associated with AMR.

In the high-potential scenario (see table 2B), we estimated that vaccination of at-risk individuals across all age groups against *E. coli*—non-diarrhogenic could avert 0.39 (0.37–0.40) million deaths and 13 (12–13) million DALYs associated with AMR in 2019. Vaccination of at-risk individuals against *K. pneumoniae* could avert 0.32 (0.31–0.34) million deaths and 14 (13–15) million DALYs associated with AMR, and vaccination against *S. aureus* could avert 0.32 (0.31–0.33) million deaths and 11 (10–11) million DALYs associated with AMR.

### Vaccine impact on AMR burden by infectious syndrome

Figure 2B shows the vaccine avertable deaths and DALYs attributable to and associated with bacterial AMR for the different infectious syndromes in 2019 at the global level in the baseline scenario. We estimated vaccine avertable mortality associated with bacterial AMR to be highest for LRIs at 0.16 (0.14–0.17) million deaths and 11 (9.6–11) million DALYs for the baseline scenario, followed by tuberculosis (TB) at 0.12 (0.11–0.13) million deaths and 4.5 (4.1–5.0) million DALYs and bloodstream infections at 0.11 (0.10–0.12) million deaths and 5.6 (5.1–6.3) million DALYs in 2019. In the high-potential scenario, vaccine avertable deaths and DALYs were highest for LRIs, BSIs and intra-abdominal infections.

**Figure 3:**
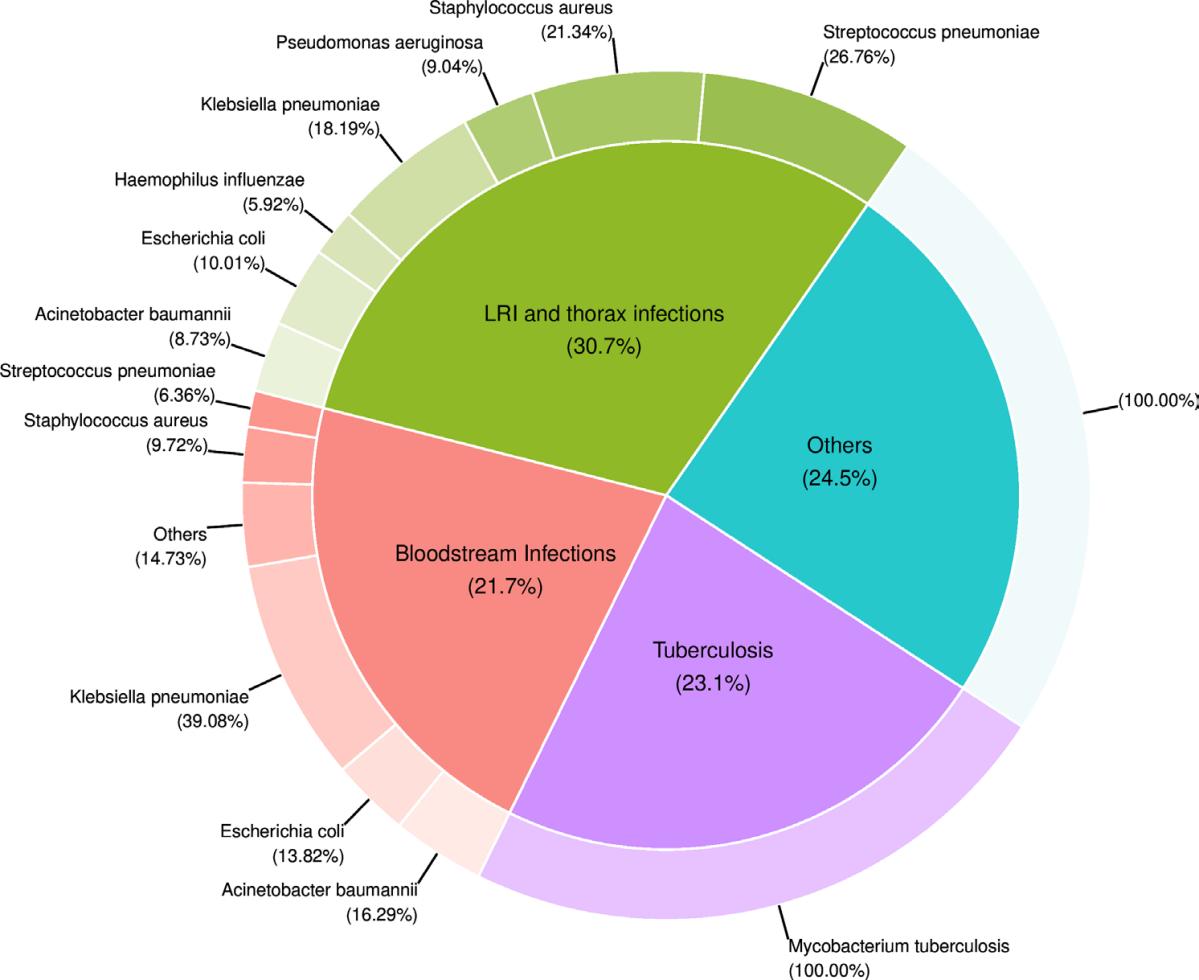
Vaccine avertable AMR burden by infectious syndrome and pathogen. Vaccine avertable deaths associated with AMR by infectious syndrome and pathogen in the baseline scenario. (“Others” include infections of bones, joints, and related organs, bloodstream infections, endocarditis and other cardiac infections, meningitis and other bacterial CNS infections, peritoneal and intra-abdominal infections, lower respiratory infections and all related infections in the thorax, bacterial infections of the skin and subcutaneous systems, typhoid fever, paratyphoid fever, and invasive non-typhoidal Salmonella spp, and urinary tract infections and pyelonephritis)

For each infectious syndrome, we stratified the vaccine avertable AMR burden for deaths and DALYs by pathogen in the baseline scenario, as shown in figure 3 and online supplemental figure A1. *S. pneumoniae*, *S. aureus* and *K. pneumoniae* account for most of the vaccine avertable AMR burden associated with LRIs. *K. pneumoniae*, *A. baumannii* and *E. coli* account for most of the vaccine avertable AMR burden associated with BSIs.

### Vaccine impact on AMR burden at the regional level

Table 3 and figure 2C show the vaccine avertable deaths and DALYs attributable to and associated with bacterial AMR at the regional levels in 2019 for the baseline scenario. We estimated the vaccine avertable burden associated with bacterial AMR to be highest in the WHO Africa region at 0.17 (0.15–0.18) million deaths and 12 (11–13) million DALYs, followed by the WHO South-East Asia region at 0.16 (0.15–0.18) million deaths and 7.5 (6.8–8.5) million DALYs in 2019. The vaccine avertable AMR burden for the WHO Africa and South-East Asia regions accounts for around two-thirds of the vaccine avertable AMR burden globally in 2019. In the high-potential scenario, we estimated that vaccines would avert an additional 0.19 (0.18–0.20) million deaths and 9.6 (8.8–11) million DALYs associated with AMR in the WHO Africa region, and 0.32 (0.30–0.33) million deaths and 11 (10–11) million DALYs associated with AMR in the WHO South-East Asia region.

**Table 3:** Vaccine avertable AMR burden globally and by WHO region

WHO region	Vaccine avertable deaths (median and 95% UI)		Vaccine avertable DALYs (median and 95% UI)	
	Associated with resistance	Attributable to resistance	Associated with resistance	Attributable to resistance
Africa	166 105 (154 785–180 343)	44 745 (40 658–49 196)	12 311 693 (11 333 145–13 343 956)	3 092 513 (2 783 222–3 396 579)
Americas	32 901 (30 020-35 892)	8824 (7 949–9 939)	1 153 608 (1 062 629–1 250 636)	295 226 (269 907–321 201)
Eastern Mediterranean	61 060 (56 445–66 784)	18 105 (16 426–20 194)	4 100 742 (3 742 727–4 559 594)	1 133 518 (1 023 025–1 258 309)
Europe	32 218 (29 145–37 168)	9721 (8 646–11 126)	944 991 (875 082–1 031 180)	291 308 (265 281–325 272)
South-East Asia	162 699 (147 461–179 566)	54 989 (47 336–64 667)	7 523 796 (6 770 514–8 458 311)	2 289 200 (2 014 438–2 684 501)
Western Pacific	58 701 (52 392–67 736)	16 569 (14 593–19 491)	1 933 726 (1 778 575–2 113 470)	508 474 (466 317–566 236)
Global	514 631 (491 550–540 336)	153 009 (144 253–165 008)	27 978 617 (26 626 506–29 377 853)	7 620 837 (7 133 393–8 046 001)

The estimates (median and 95% UIs) for vaccine avertable disease burden attributable to and associated with bacterial AMR in 2019 is presented in terms of deaths and DALYs avertable by vaccination in the baseline scenario.

## Discussion

We estimated vaccine avertable disease burden attributable to and associated with AMR for existing and new vaccines in the pipeline by pathogen, infectious syndrome and region based on the most recent, comprehensive estimates of the global burden of AMR. The AMR burden avertable by vaccination in 2019 was highest for the WHO Africa and South-East Asia regions, for LRIs, TB and BSIs by infectious syndromes, and for *M. tuberculosis* and *S. pneumoniae* by pathogen.

Our estimates show the impact of existing vaccines for pneumococcal conjugate vaccine, *H. influenzae* type b (Hib) and typhoid conjugate vaccine (TCV) on reducing AMR burden attributable to and associated with *S. pneumoniae*, *H. influenzae* and *Salmonella typhi,* respectively. We highlight the critical need to scale up existing vaccines to high and equitable immunisation coverage, and the acceleration of TCV introductions in high burden countries. Also, we show that vaccines can contribute towards preventing a significant proportion of the AMR burden for pathogens which have vaccines in late-stage clinical development with clear attributes or published PPCs or TPPs, such as for ExPEC and *M. tuberculosis*. Novel regulatory and policy mechanisms should be developed to accelerate the approval and use of these vaccines to prevent AMR. Based on the estimated high vaccine avertable burden associated with AMR for *K. pneumoniae*, *S. aureus* and *A. baumannii*, we urgently call for studies to enhance biological understanding and improve the feasibility of developing vaccines for these pathogens. For the remaining pathogens that have vaccine candidates in the early stages of clinical development or no vaccines in the pipeline, we recommend investing in vaccine development to resolve biological challenges as well as feasibility in terms of product development, market access and product implementation.

Our analysis included a baseline and high-potential scenarios. In the baseline scenario, we model vaccine delivery based on known vaccine attributes, including a defined target age group that has been immunised with a vaccine in the past, during clinical trials or identified in vaccine TPPs. In contrast, the high-potential scenario makes no assumptions about vaccine delivery and target age group and shows the highest probable vaccine impact, should there be a policy recommendation and feasibility of delivery to all who would benefit from a vaccine. We recognise that the high-potential scenario includes multiple challenges that need overcoming such as immunisation of adults and the elderly, timely immunisation to prevent nosocomial infections, vaccine efficacy in patients who are immunocompromised and with comorbidities, vaccine demand and financing.

Pan-pathogen analyses with standardised methodologies are critical to inform vaccine funding and development and should be followed up with detailed vaccine-specific analyses, considering pathogen biology and transmission, and accounting for varied disease burden patterns across the spatial and temporal scales. *H. influenzae* type b (Hib), rotavirus, pneumococcal, typhoid and influenza vaccines have been directly associated with reduction of resistance, antibiotic use and related clinical complications,^9 18–25^ while Fu *et al* modelled the global burden of drug-resistant TB avertable by a future TB vaccine.^26^

Our study has limitations. First, since we included the direct effect of vaccination but excluded indirect effect and transmission dynamics of AMR pathogens, our vaccine impact estimates on averted AMR burden are conservative. Second, our analysis focused on 15 bacterial pathogens and additional pathogens included in the GRAM project such as *Enterobacter* spp, Group B *Streptococcus*, *E. feacalis*, *Proteus* spp, *Citrobacter* spp and *Morganella* spp were excluded. However, inclusion of these pathogens appears unlikely to significantly affect our overall inferences considering that the included 15 pathogens are responsible for the majority of the AMR burden. Third, while our analysis was based on the estimates generated by the GRAM project, which represents the most comprehensive estimates of bacterial AMR burden to date, limited input data to the GRAM project especially from low-income and middle-income countries is a significant data gap that necessitates newer surveillance data and platforms to inform the updates, validity and confidence in the estimates of the GRAM project. In particular, estimates from the GRAM project for TB do not include TB associated with HIV. Fourth, we did not consider the impact of viral vaccines on reducing the AMR drivers of antibiotic misuse and overuse.^21 23 27 28^ Finally, we did not consider geographic and socioeconomic clustering of vaccination coverage, which could lead to heterogeneity in vaccination impact on lowering AMR burden with relatively less impact among subpopulations with higher risk of disease while also facing lower healthcare access including access to vaccination services.^29^

The value of vaccines in preventing AMR should be systematically considered in the decision-making process during scale-up of existing vaccines and introduction of new vaccines. Vaccines should be explicitly incorporated as tools to combat AMR into National Action Plans on AMR^30^ and National Immunisation Strategies.^31^ For new vaccines in the pipeline and future vaccines, we recommend vaccine avertable burden of AMR to be included in the full value of vaccine assessments.^32^ This evidence can support stakeholders in their decision-making process and priority setting throughout the end-to-end continuum from discovery and clinical development to investment, development, introduction and sustainability of new vaccines with equitable access.

10.1136/bmjgh-2022-011341.supp2 Supplementary data

## Data Availability

Data are available in a public, open access repository.
