# Supplementary material for: Global and regional burden of attributable and associated bacterial antimicrobial resistance avertable by vaccination: modelling study
Source: BMJ Glob Health. 2023 Jul 5;8(7):e011341. doi: 10.1136/bmjgh-2022-011341 (PMC10335446; doi:10.1136/bmjgh-2022-011341)
Supplement: Supplementary data [file bmjgh-2022-011341supp001.pdf]

Appendix

A1. Additional details of the modelling process

Estimating vaccine-avertable AMR burden of the target age group

The AMR burden data from the Global Research on Antimicrobial Resistance (GRAM) project was disaggregated by age and included the following categories – early neonatal (first week after birth), late neonatal (2-4 weeks of age), postneonatal (5 weeks to under 1-year of age), 1-4 years, 4-9 years, … , 90-94 years, and 95 years and beyond. We estimated the reduction in AMR burden in direct proportion to efficacy, coverage, target population for protection, and duration of protection of existing and potential future vaccines. We considered that immunised individuals would gain vaccine-derived immunity two weeks post-vaccination.

Estimating pre-vaccination burden for pathogens with existing vaccines

For the existing Hib and pneumococcal conjugate vaccines (PCVs), we estimated the pre-vaccination (i.e., no vaccination) burden associated and attributable to AMR in 2019 using the estimates of efficacy and coverage in 2019. We used the vaccine coverage for Hib and PCV from WHO/UNICEF Estimates of National Immunization Coverage (WUENIC) (1) and demography data from the United Nations World Population Prospects (UNWPP) (2) to estimate the vaccine coverage at the regional level. We used the vaccine efficacy estimates for the first dose, second dose, and third doses scheduled at 6, 10, and 14 weeks for the Hib (3,4) and PCV vaccines (5,6). By applying the vaccine efficacies and regional coverage to the AMR burden data in 2019, we estimated the increase in AMR burden for the counterfactual scenario of no vaccination in direct proportion to efficacy, coverage, target population for protection, and duration of protection. The global and regional coverage of typhoid conjugate vaccine and the post-vaccination impact was minimal in 2019 (7) and thereby did not warrant additional estimation for the counterfactual scenario of no vaccination.

The GRAM project estimates of AMR burden for *H. influenzae* were not stratified by serotypes. *H. influenzae* serotype b (Hib) was responsible for around 95% of all invasive *H. influenzae* disease burden among children younger than 5 years of age before the introduction of vaccines (8). By applying the 95% Hib proportion to the total *H. influenzae* burden in the counterfactual scenario of no vaccination, we estimated the vaccine-preventable proportion of Hib-specific AMR burden of the total *H. influenzae* AMR burden in 2019.

Disease type specification of the AMR burden

The GRAM project's AMR burden estimations do not differentiate between *Escherichia coli* strains. Instead, the AMR burden estimates were stratified by symptoms. Since enterotoxigenic *E. coli* (ETEC)

and enteropathogenic *E. coli* (EPEC) are the two major *E. coli* strains that cause diarrhoea, we calculated the proportional contribution of ETEC to the AMR burden due to *E. coli* causing diarrhoea and then estimated the impact of the ETEC vaccine on reducing this burden (43.97%).

Estimating the aggregated vaccine avertable burden

To estimate the aggregate estimates on the impact of the vaccines by region and by infectious syndrome, we estimated the impact of all listed vaccines as long as the effects do not overlap to avoid double counting. When there were multiple vaccines which target the same disease, infectious syndrome, and age, we chose the vaccines with greater efficacy for these estimates. However, for vaccines against *Streptococcus pneumoniae*, we used the efficacy of the existing vaccine with increased coverage that met the strategic priority on coverage and equity of Immunisation Agenda 2030.

Scenarios for vaccine avertable AMR burden

We estimated vaccine avertable AMR burden for baseline and high-potential scenarios. We recognise that the high-potential scenario is optimistic given the unanswered questions about the feasibility of producing vaccines with long-term immunity and timely delivery to populations at risk, such as patients in hospitals undergoing elective surgeries. We included the high-potential scenario to highlight the potential impact vaccines could have if challenges around vaccine development and delivery were to be resolved.

Uncertainty analysis

Our estimations account for the uncertainties around AMR burden, efficacy, and coverage. Based on data examination, we applied the lognormal distribution to the mean, the 2.5th and 97.5th percentiles of the AMR burden to generate the randomly drawn values. For vaccine efficacy and coverage, we used the truncated normal distribution. For hypothetical vaccines, we applied ± 20% to the vaccine efficacy and coverage on the vaccine profile. For existing vaccines, we used confidence intervals of the vaccine efficacy from studies and applied ± 5% to vaccine coverage (that is, coverage of existing vaccines increased in order to meet the strategic priority on coverage and equity of Immunisation Agenda 2030). When estimating the impact of the existing vaccines with current coverage (that is, based on WUENIC estimates), we only included the uncertainty in efficacy as we used the point estimates of actual coverage.

Figure A1. Vaccine avertable AMR burden by infectious syndrome and pathogen. Vaccine avertable AMR burden (deaths and DALYs averted) by infectious syndrome and pathogen in the baseline scenario. (A) Vaccine avertable deaths attributable to AMR by infectious syndrome and pathogen. (B) Vaccine avertable DALYs associated with AMR by infectious syndrome and pathogen. (C) Vaccine avertable DALYs attributable to AMR by infectious syndrome and pathogen.

(A)

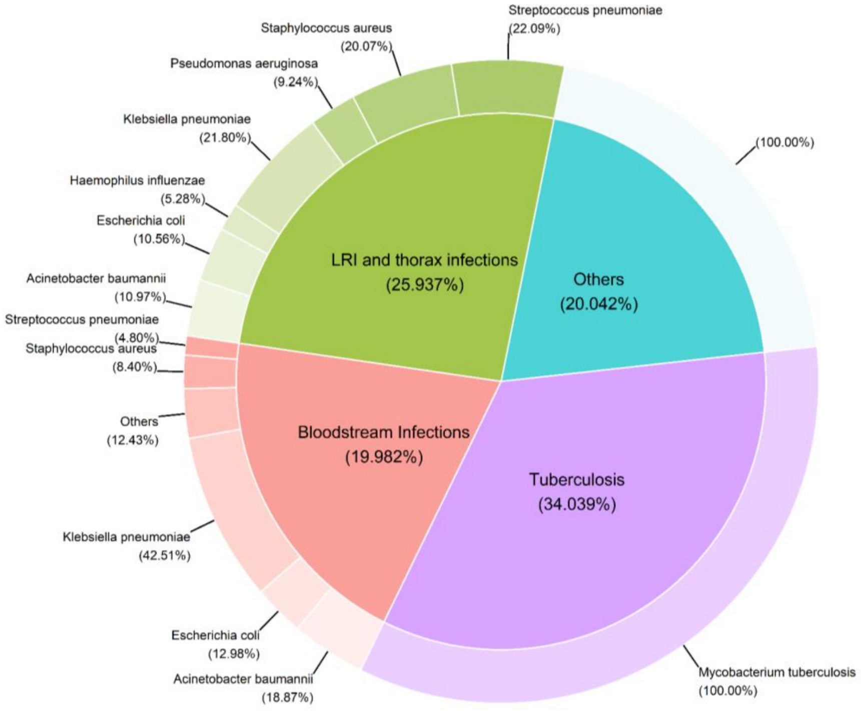

(B)

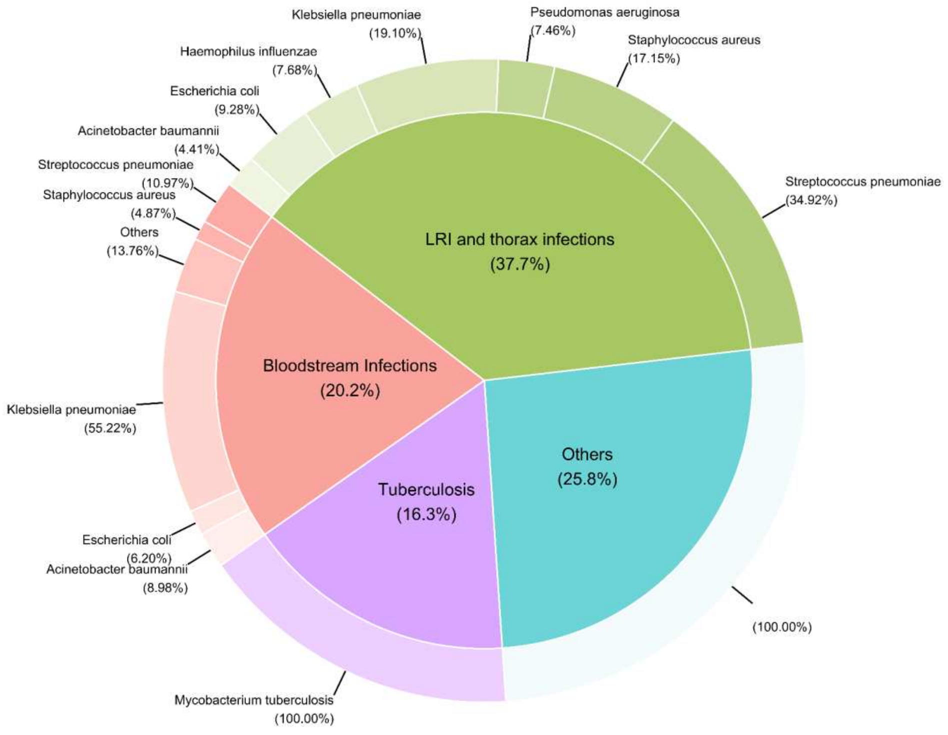
Klebsiella pneumoniae
(19.10%)
Pseudomonas aeruginosa
(7.46%)
Staphylococcus aureus
(17.15%)
Haemophilus influenzae
(7.68%)
Escherichia coli
(9.28%)
Acinetobacter baumannii
(4.41%)
Streptococcus pneumoniae
(10.97%)
Staphylococcus aureus
(4.87%)
Others
(13.76%)
Streptococcus pneumoniae
(34.92%)
LRI and thorax infections
(37.7%)
Bloodstream Infections
(20.2%)
Klebsiella pneumoniae
(55.22%)
Others
(25.8%)
Tuberculosis
(16.3%)
Escherichia coli
(6.20%)
Acinetobacter baumannii
(8.98%)
(100.00%)
Mycobacterium tuberculosis
(100.00%)

(C)

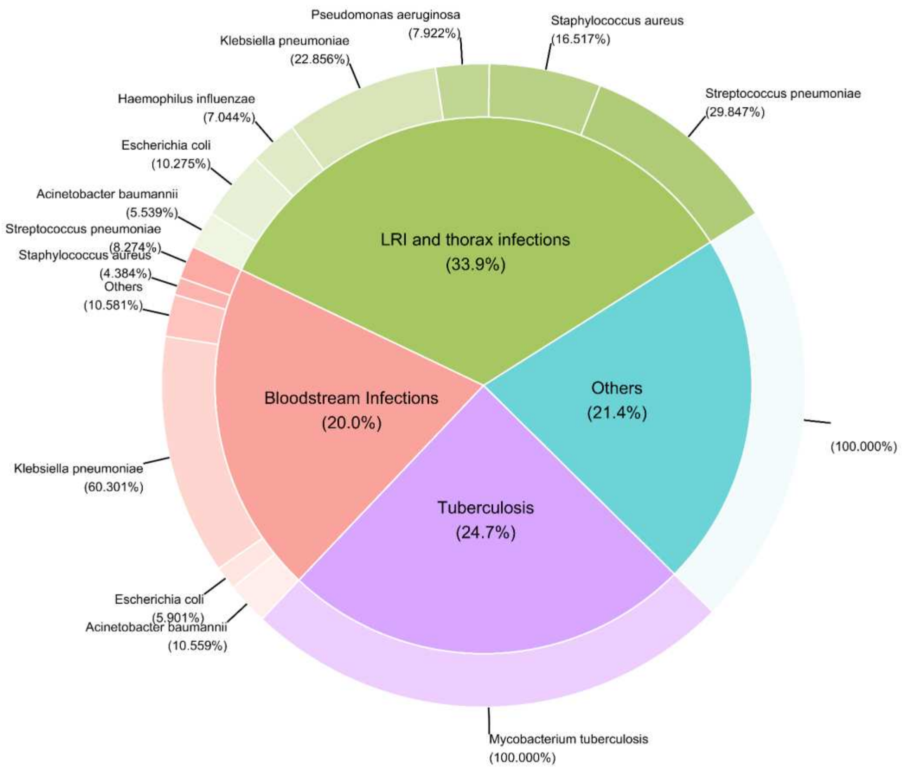
Pseudomonas aeruginosa
(7.922%)
Staphylococcus aureus
(16.517%)
Klebsiella pneumoniae
(22.856%)
Streptococcus pneumoniae
(29.847%)
Haemophilus influenzae
(7.044%)
Escherichia coli
(10.275%)
Acinetobacter baumannii
(5.539%)
Streptococcus pneumoniae
(8.274%)
Staphylococcus aureus
(4.384%)
Others
(10.581%)
LRI and thorax infections
(33.9%)
Others
(21.4%)
(100.000%)
Bloodstream Infections
(20.0%)
Klebsiella pneumoniae
(60.301%)
Tuberculosis
(24.7%)
Escherichia coli
(5.901%)
Acinetobacter baumannii
(10.559%)
Mycobacterium tuberculosis
(100.000%)

Table A1. Vaccine avertable AMR burden by WHO region, infectious syndrome, and pathogen in the baseline scenario. Vaccine avertable AMR burden (deaths and DALYs averted) by WHO region, infectious syndrome and pathogen in the baseline scenario. (A) Vaccine avertable deaths associated with AMR by WHO region, infectious syndrome, and pathogen. (B) Vaccine avertable deaths attributable to AMR by WHO region, infectious syndrome, and pathogen. (C) Vaccine avertable DALYs associated with AMR by WHO region, infectious syndrome, and pathogen. (D) Vaccine avertable DALYs attributable to AMR by WHO region, infectious syndrome, and pathogen. This table can be found in Supplemental File 2.

Bibliography

1. WHO, UNICEF. WUENIC (WHO/UNICEF Estimates of National Immunization Coverage) estimates - Vaccines monitoring system 2019 Global Summary Reference Time Series [Internet]. 2019 [cited 2023 Jun 11]. Available from: https://www.who.int/teams/immunization-vaccines-and-biologicals/immunization-analysis-and-insights/global-monitoring/immunization-coverage/who-unicef-estimates-of-national-immunization-coverage

2. United Nations. World Population Prospects 2019, United Nations Department of Economic and Social Affairs Population Division [Internet]. 2019 [cited 2019 Nov 18]. Available from: https://population.un.org/wpp/

3. Griffiths UK, Clark A, Gessner B, Miners A, Sanderson C, Sedyaningsih ER, et al. Dose-specific efficacy of Haemophilus influenzae type b conjugate vaccines: a systematic review and meta-analysis of controlled clinical trials. Epidemiol Infect. 2012 Aug;140(8):1343–55.

4. Obonyo CO, Lau J. Efficacy of Haemophilus influenzae type b vaccination of children: a meta-analysis. Eur J Clin Microbiol Infect Dis. 2006 Feb;25(2):90–7.

5. Lucero MG, Dulalia VE, Nillos LT, Williams G, Parreño RAN, Nohynek H, et al. Pneumococcal conjugate vaccines for preventing vaccine-type invasive pneumococcal disease and X-ray defined pneumonia in children less than two years of age. Cochrane Database Syst Rev. 2009 Oct 7;(4):CD004977.

6. Mahon BE, Hsu K, Karumuri S, Kaplan SL, Mason EO, Pelton SI, et al. Effectiveness of abbreviated and delayed 7-valent pneumococcal conjugate vaccine dosing regimens. Vaccine. 2006 Mar 24;24(14):2514–20.

7. Neuzil KM, Pollard AJ, Marfin AA. Introduction of typhoid conjugate vaccines in Africa and Asia. Clin Infect Dis. 2019 Feb 15;68(Suppl 1):S27–30.

8. WHO. Immunological Basis for Immunization Series: Module 9: Haemophilus influenzae type b [Internet]. 2007 [cited 2022 Apr 22]. Available from: https://www.who.int/publications/i/item/who-immunological-basis-for-immunization-series-module-9-haemophilus-influenzae-type-b
